# Infrahepatic Inferior Vena Cava Clamping Reduces Blood Loss during Liver Transection for Cholangiocarcinoma

**DOI:** 10.1155/2021/1625717

**Published:** 2021-08-26

**Authors:** Natwutpong Leeratanakachorn, Vor Luvira, Theerawee Tipwaratorn, Suapa Theeragul, Apiwat Jarearnrat, Attapol Titapun, Tharatip Srisuk, Supot Kamsa-ard, Ake Pugkhem, Narong Khuntikeo, Chawalit Pairojkul, Vajarabhongsa Bhudhisawasdi

**Affiliations:** ^1^Department of Surgery, Faculty of Medicine, Khon Kaen University, 123 Mittraparp Road, Meaung District, Khon Kaen 40002, Thailand; ^2^Department of Epidemiology and Biostatistics, Faculty of Public Health, Khon Kaen University, 123 Mittraparp Road, Meaung District, Khon Kaen 40002, Thailand; ^3^Department of Pathology, Faculty of Medicine, Khon Kaen University, 123 Mittraparp Road, Meaung District, Khon Kaen 40002, Thailand

## Abstract

**Background:**

Major hepatectomy is the mainstay of the treatment for cholangiocarcinoma. Infrahepatic inferior vena cava (IVC) clamping is an effective maneuver for reducing blood loss during liver transection. The impact of this procedure on major hepatectomy for cholangiocarcinoma is unknown. This study evaluated the effect of infrahepatic IVC clamping on blood loss during liver transection.

**Methods:**

Clinical and pathological data were collected retrospectively for 116 cholangiocarcinoma patients who underwent major hepatectomy between January 2015 and December 2016, to investigate the benefit of infrahepatic IVC clamping. Two of five surgeons adapted the policy performing infrahepatic IVC clamping during liver transection in all cases. Patients, therefore, were divided into those (*n* = 39; 33.6%) who received infrahepatic IVC clamping during liver transection (C1) and those (*n* = 77; 66.4%) who did not (C0).

**Results:**

The patients' backgrounds, operative parameters, and extent of hepatectomy did not differ significantly between the 2 groups, except for gender. A significantly lower blood loss (*p* = 0.028), blood transfusion (*p* = 0.011), and rate of vascular inflow occlusion requirement (*p* < 0.001) were observed in the C1 group. The respective blood losses in the C1 group and the C0 group were 498.9 (95% CI: 375.8-622.1) and 685.6 (95% CI: 571-800.2) millilitres.

**Conclusions:**

The current study found infrahepatic IVC clamping during liver transection for cholangiocarcinoma reduces blood loss, blood transfusion, and rate of vascular inflow occlusion requirement.

## 1. Introduction

Cholangiocarcinoma (CCA) is a considerably rare primary tumor of the liver [1, 2], for which the mainstay of treatment is major surgical resection [1, 3–5]. Excessive blood loss and perioperative blood transfusion have a negative impact on postoperative complications and consequently lead to worsening of the patients' overall survivals [6, 7]. Limiting blood loss, therefore, is the major issue for hepatic resection.

Bleeding during hepatectomy is generally from the inflow system (hepatic arteries and portal veins) and outflow system (hepatic veins). With the control of vascular inflow, the main source of blood loss comes from backflow from the hepatic veins. Various methods have been applied to reduce blood flow through the hepatic veins, including control of the central venous pressure (CVP) [8], exclusion of hepatic veins [9], and infrahepatic inferior vena cava (IVC) clamping [10].

Infrahepatic IVC clamping has been proved to reduce blood loss during hepatectomy. There have been many randomized controlled trials showing the benefit of this procedure for patients with primary and secondary tumor of the liver [11–16], but none of them focusing on only cholangiocarcinoma, which requires major hepatectomy. This study, therefore, is aimed at evaluating the benefits of infrahepatic IVC clamping for reducing blood loss during hepatectomy for cholangiocarcinoma patients.

## 2. Materials and Methods

### 2.1. Patients

This study was a comparative study. We retrospectively reviewed medical records and pathological data collected prospectively for all histologically confirmed CCA patients who underwent hepatectomy. From January 2015 to December 2016, curative-intent hepatic resection was performed for 116 patients with CCA at Srinagarind Hospital, Khon Kaen University. During that time, two out of five surgeons routinely performed IVC clamping, while the other three surgeons did not. This divided patients into two distinct groups: the IVC clamping (C1) and no clamping groups (C0). We analyzed the outcomes of patients of these two groups.

#### 2.1.1. Preoperative Preparation

Patients with CCA diagnosed by cross-sectional imaging received the common protocol which included the following:

1. Evaluation of resectability with cross-sectional imaging: computed tomography, magnetic resonance imaging, or both
2. Blood examination including complete blood count, liver function test, and coagulogram
3. Preoperative biliary drainage in patients presenting with jaundice with the aim to reduce serum total bilirubin to below 10 mg/dl
4. Portal vein embolization if the expected future liver remnant was not sufficient

Criteria for resectability included (1) good performance status (ECOG 0-1), (2) absence of distant organ metastasis, (3) absence of para-aortic, aortocaval, and retrocaval lymphadenopathy, and (4) adequate expected future liver remnant after surgery.

#### 2.1.2. Operative Procedure

The type of hepatic resection was determined by the location and extent of the tumor. Surgeons preferred major hepatic resection to optimize the surgical margin. Minor hepatic resection was performed only in patients with limited future liver function. Liver parenchyma transection techniques depended on the surgeon's preference. Vascular inflow occlusion was not routinely performed during liver transection. If adequate bleeding control was not achieved during liver transection, the surgeons were allowed to perform vascular inflow occlusion.

**Figure 1:**
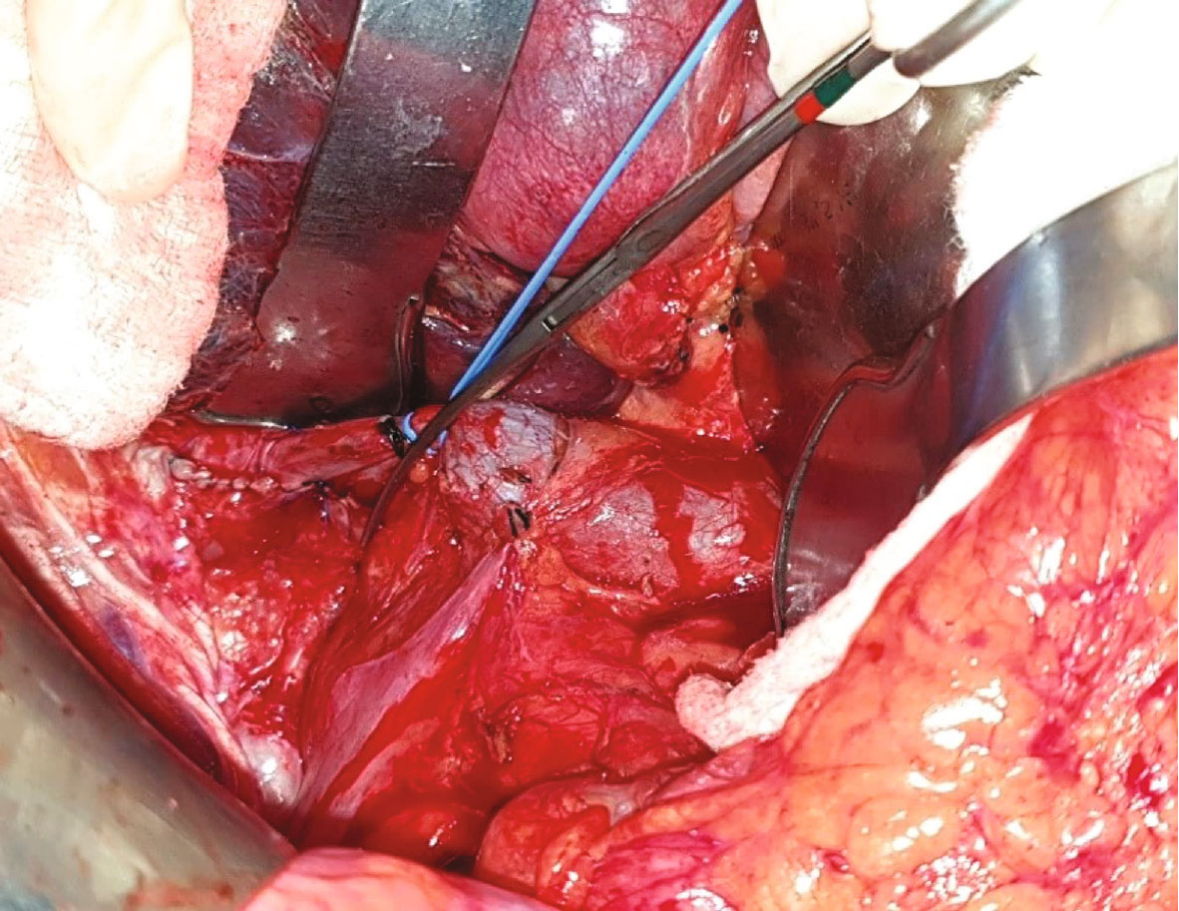
Infrahepatic IVC clamping.

Regarding the IVC clamping procedure, we dissected completely around the IVC and then hung with a vascular loop. We used a vascular clamp to perform complete infrahepatic IVC clamping during parenchymal transection (Figure 1). All surgical specimens were sent to the Department of Pathology.

### 2.2. Outcome Variables

The primary outcome of this study was intraoperative blood loss, which was measured by the amount of blood gathered in a suction container and from the sponges and gauzes calculated by its weight, then subtracted by the volume of fluid irrigation. We also investigated the amount of blood transfusion, rate of requirement of vascular inflow occlusion, postoperative stay, postoperative mortality, and complications as secondary outcomes.

### 2.3. Statistical Analysis

Descriptive analyses were performed and reported as appropriate (i.e., mean, standard deviation: SD, median, interquartile range; IQR, number and percent). Continuous data were analyzed using Student's *t*-test. Categorical data were compared with the Pearson *χ*^2^ test. Survival analysis was performed using the Kaplan–Meier analysis. A *p* value of less than 0.05 was considered statistically significant. All statistical analyses were performed using STATA version 13 (Lakeway, TX, USA).

### 2.4. Ethical Consideration

The Institutional Review Board, Office of Human Research Ethics, Khon Kaen University, reviewed and approved this study (HE601032).

## 3. Results

### 3.1. Demographic Data

There were 77 patients (66.4%) in the no clamp group (C0) and 39 patients (33.6%) in the IVC clamp group (C1). The median age of the study population was 63 (+/-9.5) years, and male patients outnumbered female (62.1 versus 37.9%). The proportion of male patients was higher in the C0 group (67.5%) compared with the C1 group (51.3%). Most tumors were extrahepatic CCA (55.2%), and the most common morphology was intraductal tumor (61.2%). There were no cirrhotic patients included in this study. Otherwise, there were no statistically significant differences between both groups in baseline preoperative data (Table 1).

### 3.2. Operative Outcomes

**Figure 2:**
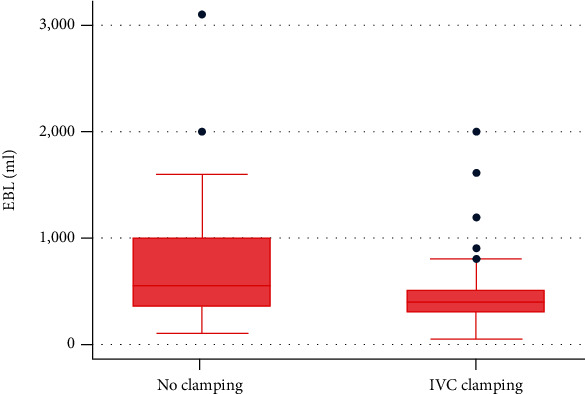
Comparison of mean estimated blood loss (EBL) between the IVC clamping and no IVC clamping groups.

The operative parameters are shown in Table 2. Most of the patients underwent right-sided hepatectomy (67.2%). Bile duct resection and vascular resection were performed in 55.2% and 6.9%, respectively. There was no difference of proportion of the bile duct and vascular resection rate between the two groups. The median IVC clamping time in the C1 group was 40 (+/-30) minutes. The maximal and minimal CVP during liver parenchymal transection was not different between the two groups. The mean intraoperative blood loss was significantly lower (*p* = 0.028) in the IVC clamping group at 498.9 (95% CI: 375.8-622.1) ml, while that in the no clamping group was 685.6 (95% CI: 570.9-800.2) ml (Figure 2). The amount of blood transfusion was concordantly lower in the IVC clamping group at 64  (95% CI: 15.0-113.6) ml while that in the no clamping group was 171 (95% CI: 104.2-237.6) ml. We also found that the rate of vascular inflow occlusion was significantly lower in the C1 group than in the C0 group, at 5.13% vs. 57.4% (*p* = 0.011).

### 3.3. Postoperative Outcomes

The postoperative outcomes are shown in Table 3. There was no 30-day mortality in this cohort. There were three patients with 60-day mortality. Patients died on postoperative days 21, 37, and 45 from bleeding false aneurysm of right hepatic stump, severe pneumonia, and severe postoperative liver failure, respectively. All of these were in the C0 group.

The postoperative complication rate and postoperative hospital stay were not different between the two groups. There were slightly higher rates of postoperative liver failure and wound complication in the C1 group.

With the median follow-up time of 1,234 days, the median survival time was 1,302 days (95% CI: 884-1654 days) such that the respective 1- and 3-year overall survival rates were 80.9% (95% CI:72.4–86.9) and 55.5% (95% CI:45.9–64.1). There was no difference in patient survival between the C0 and C1 groups (*p* = 0.889).

## 4. Discussion

Many studies have shown the benefit of infrahepatic IVC clamping for reducing blood loss during hepatic resection [17, 18]. This study supports this effect also in hepatic resection for cholangiocarcinoma. We, moreover, found that infrahepatic IVC clamping was associated with a lower amount of blood transfusion and a lower rate of vascular inflow occlusion requirement during liver transection. Until now, there has been no study investigating the effects of infrahepatic IVC clamping focusing only on hepatic resection for cholangiocarcinoma.

Blood loss during liver transection depends on many factors including status of the liver, patient co-morbidities, type of tumor, tumor extension, surgical procedures, anesthesiologist team, surgical devices, and, importantly, surgeon factors, which include individual surgical skill, hepatic resection experience, and surgical technique [19]. Our finding that infrahepatic IVC clamping reduces blood loss and, subsequently, reduces blood transfusion is quite straightforward. Since the main source of blood loss during liver transection can be simply divided into inflow and backflow, controlling either side or both of these two would result in less blood loss. This could be applied as a universal principle for all hepatic resections. Our findings are compatible with previous reports, which found that infrahepatic IVC clamping was associated with lower blood loss and blood transfusion [17, 18]. Despite the similarity of the main findings of these studies, there is variety among the studies of the included participants and the magnitude of the benefit of IVC clamping regarding the disease. The factors showing an augmented effect of the IVC clamp were anatomical hepatic resection, exposing hepatic vein during liver transection [15], resection using anterior hepatectomy technique [14], the degree of severity of liver cirrhosis [15], and being unable to maintain low CVP during liver transection [10, 11, 16]. Our study evaluated the benefit of infrahepatic IVC clamping focusing on patients with cholangiocarcinoma, which gave some unique features of hepatic resection, including (i) the requirements of anatomical major liver resection; (ii) being quite simple but having a large transection surface; (iii) being noncirrhotic but, sometimes, having a tense liver from biliary obstruction; iv) the plan: the resected lobe is usually already de-vascularized; and (v) some limiting of the exposure because the bile duct remains intact during liver transection [16]. A previous study investigated the effects of infrahepatic IVC clamping in participants with primary liver tumor but failed to demonstrate the benefit in the nonhepatocellular carcinoma subgroup, because of the low number of cholangiocarcinoma participants [15], while our study was able to demonstrate this effect. We, moreover, found that vascular inflow occlusion could be omitted if we had good control of the backflow using an infrahepatic IVC clamping technique. While most centers routinely perform vascular inflow occlusion during liver transection [11, 13, 15], our center's policy is to perform this procedure in selected cases, depending on the performing surgeon. We, therefore, add the new knowledge of reducing the rate of vascular inflow occlusion by infrahepatic IVC clamping.

As for safety, infrahepatic IVC clamping might theoretically cause various degrees of pulmonary embolism [12] and decreasing venous return, which might result in significant hypotension, various degrees of renal damage, and mortality [9]. However, as in previous reports [18], our results reveal that infrahepatic IVC clamping causes no significant influence on such complications as these, especially cardiovascular and renal complications. The reason that may account for this is that most of our patients did not have low CVP during liver resection. We, moreover, found that intraoperative CVP and fluid amount usage did not differ between the two groups. These findings were compatible with previous meta-analysis [18]. The explanations for these include the following: (i) During infrahepatic IVC clamping, the cardiovascular parameters were maintained from blood flow through other routes (i.e., subphrenic, adrenal, lumbar, and retroperitoneal veins), and they might deliver some adrenal hormone into the circulation [13, 18]. (ii) In the IVC clamping group, only 5.13 percent of the patients required vascular inflow occlusion. Without vascular inflow occlusion, blood was allowed to flow via the viable lobe of the liver, maintaining venous return. (iii) Although there was no difference in total fluid usage between the groups during the whole operative time, it might have a difference according to the phase of the operation. The surgeons, who are aware of possible backflow bleeding from hepatic veins, usually restrict fluid intake before and during the hepatic-transection phase, and use vasopressor, rather than fluid, to just keep marginal urine output, then allow aggressive fluid resuscitation to normalize blood pressure and urine output after the transection phase [19].

Tumor-related survival has been found to be related to the amount of operative blood loss, from the disturbance to immune function. While a previous study has reported that the amount of blood loss significantly affects the survival of patients with HCC [6], we found no difference in long-term survival of patients between the infrahepatic IVC clamping and nonclamping groups. The reasons that may account for this difference include the following: (i) there was little difference in the amount of blood loss between groups and (ii) unlike hepatocellular carcinoma, cholangiocarcinoma is not an immunologic-sensitive tumor [20]. Nevertheless, it indicates that there was no difference in the severity of disease between the two groups in our study.

Given the low morbidity rate and the benefit of reducing blood loss, we recommend attempting to control backflow bleeding in every liver transection. There are many techniques, from easiest to hardest, including controlling CVP [8], infrahepatic IVC clamping [10], total or selective hepatic vascular exclusion, and hepatic vascular exclusion with preservation of caval flow [9]. However, these techniques require, at least, preoperative evaluation of the cardiovascular reserve of the patients and intensive monitoring intraoperatively, since such procedures as these may cause some degree of cardiovascular disturbance. In the worst situation, these would precipitate underlying ischemic heart disease, leading subsequently to intraoperative mortality. Controlling CVP requires good teamwork by anesthesiologists and its benefit on reducing blood loss is inferior to the surgeon-dependently infrahepatic IVC clamping technique [13, 16]. Compared with controlling CVP and total hepatic vascular exclusion, infrahepatic IVC clamping seems to cause fewer hemodynamic disturbances. Moreover, IVC clamping is less technically demanding compared with hepatic vascular exclusion with preservation of caval flow, in which dissection of hepatic veins is required [14].

To the best of our knowledge, this is the first study on the effects of infrahepatic IVC clamping focusing on hepatic resection for cholangiocarcinoma. This study must, however, be interpreted carefully. While most of the operative and pathology data were recorded prospectively, the review was performed retrospectively, so it may introduce some selection bias and inaccuracy of the estimated amount of blood loss, which was the primary outcome. Moreover, owing to the nature of this quasiexperimental study, the decision to perform an infrahepatic IVC clamp did not rely on random assignment. The operating surgeon inevitably has an influence on all outcomes. However, when compared with a previous study by Kato et al. [11], the amounts of blood loss for both groups in both studies seem to be quite similar. This may indicate the reliability of such a quasiexperimental study.

## 5. Conclusions

In conclusion, this study provides evidence that infrahepatic IVC clamping can reduce intraoperative blood loss, blood transfusion volume, and rate of vascular inflow occlusion. This technique should be used as an adjunct to the available techniques for controlling backflow bleeding for hepatic resection for cholangiocarcinoma. However, a randomized controlled trial is required to confirm these results in cholangiocarcinoma patients.

## Figures and Tables

**Table 1:** Baseline characteristics.

Variables	IVC clamping (*n* = 39)		No IVC clamping (*n* = 77)	
	*n* (%) or median (IQR)		*n* (%) or median (IQR)	
*Gender*				
Male	20	(51.3%)	52	(67.5%)
Female	19	(48.7%)	25	(32.5%)
*Characteristics of lesions*				
Location				
Intrahepatic	14	(35.9%)	38	(49.4%)
Perihilar	25	(64.1%)	39	(50.6%)
Gross morphology				
Mass forming	2	(5.1%)	10	(13.0%)
Periductal infiltration	12	(30.8%)	21	(27.3%)
Intraductal growth	25	(64.1%)	46	(59.7%)
*Preoperative laboratory investigation*				
Cholesterol	123	(57)	139	(54)
Total bilirubin	1.2	(2.3)	0.9	(2.1)
AST	298	(276)	205	(289)
ALT	201	(245)	148	(204)
ALP	119.5	(119)	97	(87)
Albumin	2.7	(0.8)	2.8	(0.8)
INR	1.13	(0.19)	1.13	(0.21)
Creatinine	0.81	(0.45)	0.89	(0.28)

**Table 2:** Operative parameters.

Variables	IVC clamping (*n* = 39)		No IVC clamping (*n* = 77)		*p* value
	*n* (%) or mean (95% CI)		*n* (%) or mean (95% CI)		
Procedure					0.747
Right-sided hepatectomy	28	(71.8%)	50	(64.9%)	
Left-sided hepatectomy	10	(25.6%)	24	(31.2%)	
Sectionectomy	1	(2.6%)	3	(3.9%)	
Bile duct resection	24	(61.5%)	40	(51.9%)	0.327
Vascular resection	3	(7.7%)	5	(6.5%)	
CVP max (mmHg)	12.5	(11.6-13.4)	12.6	(11.9-13.4)	0.8574
CVP min (mmHg)	6.26	(5.5-7.0)	6.25	(5.7-6.8)	0.9835
Vascular inflow occlusion	2	(5.13%)	44	(57.1%)	<0.001
Blood loss (ml)	498.9	(375.8-622.1)	685.6	(570.9-800.2)	0.028
Blood transfusion (ml)	64.3	(15.0-113.6)	170.9	(104.2-237.6)	0.011
Fluid usage (ml)	3059.7	(2694.9-3424.5)	3086.6	(2720.4-3452.7)	0.9259

**Table 3:** Postoperative outcomes.

Variables	IVC clamping (*n* = 39)		No IVC clamping (*n* = 77)		*p* value
	*n* (%) or mean (95% CI)		*n* (%) or mean (95% CI)		
Overall morbidity	17	(43.6%)	28	(36.4%)	0.451
*Hepatobiliary complications*					0.313
Posthepatectomy liver failure	8	(20.5%)	6	(7.8%)	
Bile leakage	1	(2.6%)	3	(3.9%)	
Stricture/cholangitis	0	(0%)	1	(1.3%)	
Transient hyperbilirubinemia	2	(5.1%)	7	(9.1%)	
*General complications*					
Wound complication	8	(20.5%)	9	(11.7%)	0.204
Pulmonary embolism	0	(0%)	1	(1.3%)	0.475
Pneumonia	0	(0%)	3	(3.9%)	0.212
Cardiac complication	1	(2.6%)	6	(7.8%)	0.264
Acute kidney injury	1	(2.6%)	1	(1.3%)	0.621
Postoperative stay (days)	13.4	(11.5-15.3)	12.9	(11.1-14.8)	0.76
*Cholesterol*					
Postoperative day 1	133.8	(122.5-145.2)	133.9	(125.3-142.4)	1.00
Postoperative day 3	108.7	(99.5-117.9)	110.3	(103.6-117.0)	0.79
Postoperative day 5	101.5	(94.3-108.7)	100.8	(94.3-107.2)	0.89
*Serum albumin*					
Postoperative day 1	3.0	(2.9-3.1)	3.0	(2.9-3.1)	0.65
Postoperative day 3	2.9	(2.8-3.1)	2.9	(2.8-2.9)	0.46
Postoperative day 5	2.9	(2.7-3.0)	2.9	(2.8-2.9)	0.96
*Total bilirubin*					
Postoperative day 1	2.7	(1.9-3.5)	3.1	(2.2-4.1)	0.58
Postoperative day 3	2.4	(1.6-3.1)	2.7	(1.8-3.6)	0.64
Postoperative day 5	2.5	(1.7-3.3)	2.7	(1.7-3.6)	0.82
*Alanine aminotransferase*					
Postoperative day 1	329.4	(236.7-422.2)	269.8	(216.3-323.2)	0.23
Postoperative day 3	170.0	(136.8-203.3)	170.6	(137.7-203.5)	0.98
Postoperative day 5	95.6	(72.2-118.9)	89.2	(72.1-106.4)	0.66
*Aspartate aminotransferase*					
Postoperative day 1	429.7	(328.9-530.6)	351.4	(282.2-420.6)	0.20
Postoperative day 3	145.3	(93.8-196.9)	124.1	(102.8-145.3)	0.37
Postoperative day 5	54.0	(41.6-66.4)	55.7	(48.1-63.4)	0.81
*International normalized ratio* (*PT/INR*)					
Postoperative day 1	1.3	(1.27-1.4)	1.3	(1.26-1.34)	0.35
Postoperative day 3	1.4	(1.3-1.5)	1.4	(1.35-1.45)	0.63
Postoperative day 5	1.4	(1.33-1.5)	1.4	(1.34 -1.49)	0.90
*Postoperative mortality*					
30 days	0	(0%)	0	(0%)	
60 days	0	(0%)	3	(3.9%)	
*Survival (95% CI)*					0.8891
Median (day)	1,257	(792-1,654)	1,351	(800-1,641)	
1-year survival	79.5%	(63.1-89.1)	81.6%	(70.9-88.7)	
3-year survival	53.9%	(37.1-67.9)	56.3%	(44.4-66.7)	

## Data Availability

The patient data used to support the findings of this study are included within the article.
